# “Holographic” Autostereoscopic Displays: A Perspective on Their Technology and Potential Impact in Chemistry

**DOI:** 10.1002/chem.202301746

**Published:** 2023-09-14

**Authors:** Dennis Svatunek

**Affiliations:** ^1^ Institute of Applied Synthetic Chemistry TU Wien Getreidemarkt 9 1060 Vienna Austria

**Keywords:** chemistry education, 3D, geometries, displays, molecules

## Abstract

The three‐dimensional arrangement of atoms in molecules is essential for understanding their properties and behavior. Traditional 2D representations and digital 3D models presented on 2D media often fall short in conveying the complexity of molecular structures. Autostereoscopic displays, often marketed as “holographic” displays, pose a potential solution to this challenge. These displays, with their multi‐view and single‐view configurations, promise to advance chemistry education and research by offering accurate 3D representations with depth and parallax. In this perspective, I delve into the possibilities and limitations of autostereoscopic displays in chemistry, discussing the underlying technology and potential applications, from research to teaching and science communication. Multi‐view autostereoscopic displays excel in facilitating collaborative work by enabling multiple viewers to simultaneously perceive the same 3D structure from different angles. However, they currently suffer from low resolution and high cost, which could limit their immediate widespread adoption. Conversely, single‐view autostereoscopic displays with eye‐tracking, while limited to one viewer at a time, provide higher resolution at a lower cost, thus suggesting that they might become the technology of the future given the balance of price to performance. Despite current limitations, autostereoscopic displays possess undeniable potential for shaping the future of chemistry education and research.

## Graphical abstract

**Adding dimensions to chemistry**: I explore the potential of “holographic” autostereoscopic displays in chemistry research and education. This perspective delves into how this emerging technology could enhance the understanding of complex molecular structures, offering a more immersive and intuitive learning and research experience.

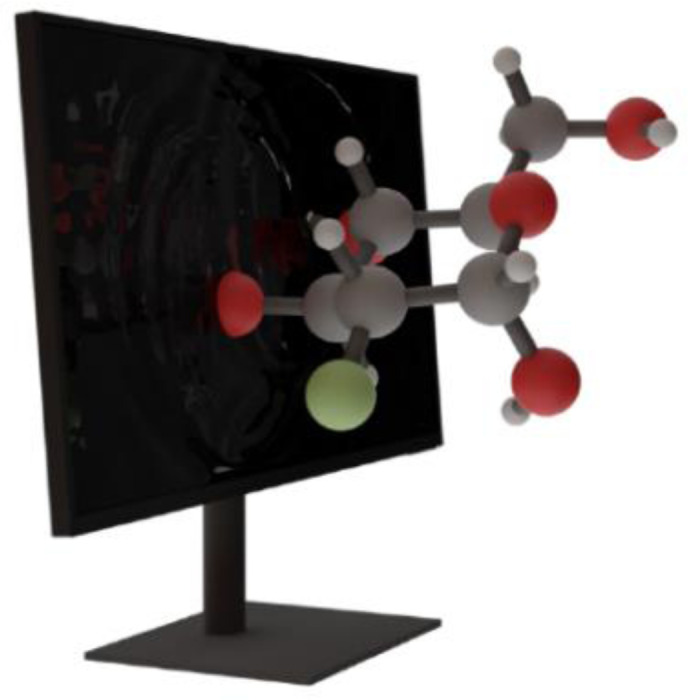

## Introduction

Chemistry relies heavily on the 3D arrangement of atoms in molecules, which governs their properties and behavior. Despite this, 2D representations are commonly used in chemical communication and education. The primary reason for this is that they allow for better visualization of molecular connectivity and can be generated more easily than 3D models. While 2D representations can provide basic information about the 3D shape of a molecule, chemists are often tasked with mentally constructing the full 3D structure from these representations. This skill is essential in many areas of chemistry and is therefore a crucial component of chemical education. To aid in this process, digital 3D models are often utilized to provide a more accurate and comprehensive visualization of molecular structure. Advances in computational chemistry have also led to sophisticated yet easily accessible software that can predict the 3D structure of molecules based on their 2D representations, greatly facilitating the study and design of new molecules with desired properties. Despite the limitations of 2D representations, they remain an important tool in chemical communication, and the ability to mentally visualize the 3D structure from these representations is a vital skill for chemists.

To gain a thorough understanding of the properties and reactivity of molecules in chemistry and biochemistry, precise 3D geometries are essential. In computational chemistry, various simulation methods are utilized to generate accurate 3D models of molecular structures. Furthermore, 3D structures can also be obtained through techniques such as X‐ray crystallography or cryogenic electron microscopy.

Despite the prevalence of 3D models in chemistry, they are typically presented using 2D media such as paper or computer screens. This projection of a 3D object onto a 2D surface can result in the loss of critical information, particularly depth and parallax. While techniques such as perspective or fog can be employed to mitigate the loss of 3D depth information, no effective solution for parallax currently exists.

Efforts have been made to provide 3D views of digital molecular models, with examples including the red‐blue anaglyph and stereo view in PerkinElmer's Chem3D. However, to my knowledge, these functionalities are rarely used. While virtual reality (VR) headsets can provide a highly accurate representation of the 3D shape of molecules,^[1]^ their use is hindered by the fact that they are cumbersome to use and limited to one person at a time, making seamless implementation into existing teaching and research processes challenging.

Autostereoscopic displays offer a robust 3D representation of molecular structures, complete with depth and parallax, enhancing the visual experience. These displays can integrate seamlessly into research workflows and pedagogical practices. Given the rise of commercially available options, both in multi‐view configurations for collaborative settings and single‐view designs with eye‐tracking for individual use, it becomes imperative to evaluate the promise and constraints of autostereoscopic technology in the context of chemistry research and teaching. This exploration considers the current state of the technology, its distinct advantages, potential challenges, and viable alternative solutions.

## Autostereoscopic Displays: Technology

Autostereoscopic displays utilize an array of optical elements, such as lenticular lenses or parallax barriers, to project distinct images to each eye.^[2]^ By creating different views of the same object and directing them towards each eye, the brain is able to interpret the images as 3D. This allows for the perception of 3D images without the need for special glasses.

Autostereoscopic displays can be classified into two categories: single view and multi‐view displays. Single view displays project only one set of views to the observer‘s eyes, typically from a fixed position. This can limit the ability to perceive the object from different angles and thus experience parallax. However, modern single view systems may include eye tracking software that adjusts the displayed view based on the position of the viewer, thus creating parallax.^[3]^

**Figure 1:**
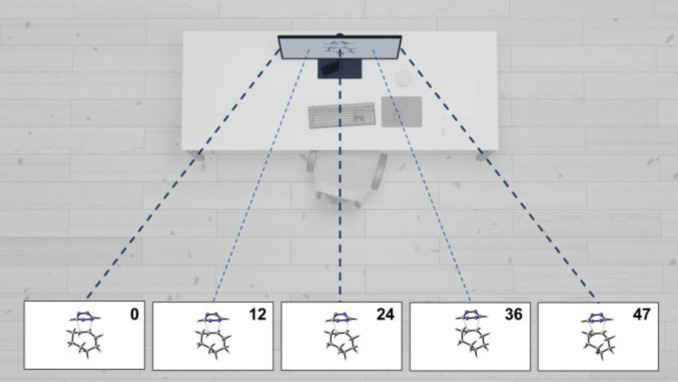
Concept of multi‐view autostereoscopic display technology showcasing 48 distinct views. View 0, positioned on the left, presents the object from a left‐sided perspective, while view 47, located on the right, displays the object as seen from the right side.

In contrast, multi‐view autostereoscopic displays generate multiple sets of views that are projected at different viewing angles, providing a wider range of perspectives and enabling the viewer to move around the object being displayed within the viewing angle, creating a sense of parallax (Figure 1). This makes multi‐view displays more immersive and useful in collaborative settings where each observer experiences their own view. However, generating multiple views requires more complex optics which is challenging and expensive to produce. Furthermore, rendering multiple distinct viewpoints demands enhanced computing power, and distributing the image across various viewing angles significantly diminishes the resolution for each individual perspective.

Multi‐view autostereoscopic displays have recently become commercially available.^[4]^ Additionally, this year saw the introduction of laptops equipped with single‐view autostereoscopic displays paired with eye‐tracking.^[3c]^ My familiarity with this technology, gained over previous years, shapes the perspective I share in this article. For an account of my experiences, refer to the Supporting Information.

**Figure 2:**
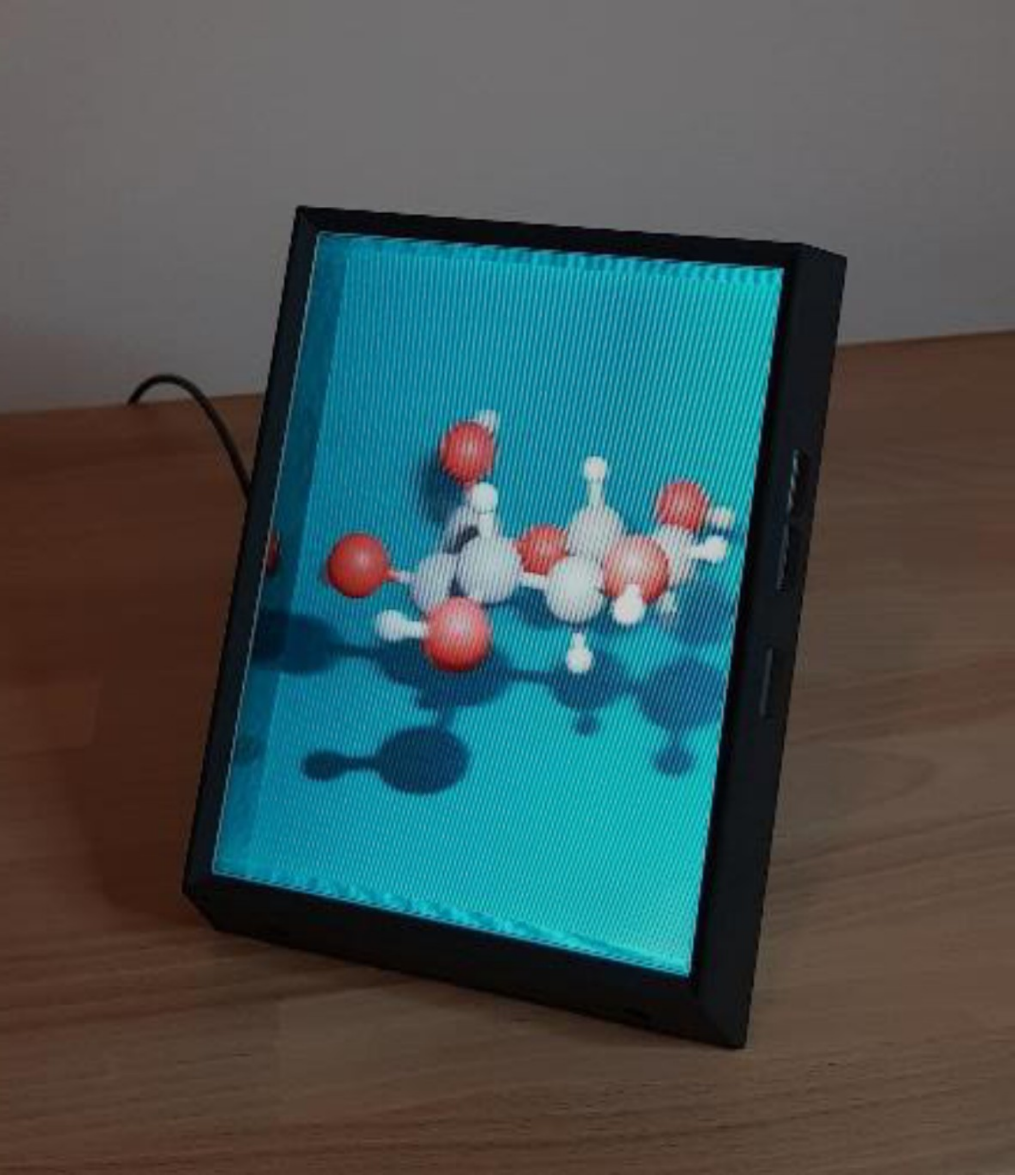
Visualization of a glucose molecule on a compact multi‐view autostereoscopic display. The image reveals artifacts stemming from the employed parallax barriers, but these artifacts are notably less pronounced when viewed directly with the naked eye.

Figure 2 showcases the 3D structure of glucose as depicted on a multi‐view autostereoscopic display. An accompanying video in the supporting material emphasizes the perceivable parallax with this setup. Presenting such chemical structures on these displays can be accomplished using several available software packages. For instance, an atomic blender plugin allows for the importation of a PDB file into the Blender 3D modeling software, and an additional plugin renders the multiple views tailored for this specific type of autostereoscopic display. With the release of version 2.4, PyMOL, a widely recognized molecular visualization tool, introduced native capabilities to present the viewport on certain multi‐view autostereoscopic displays.^[5]^ Numerous plugins are available for platforms like Unreal Engine and Unity, both of which stand as prominent tools in the realm of scientific 3D visualization.

**Figure 3:**
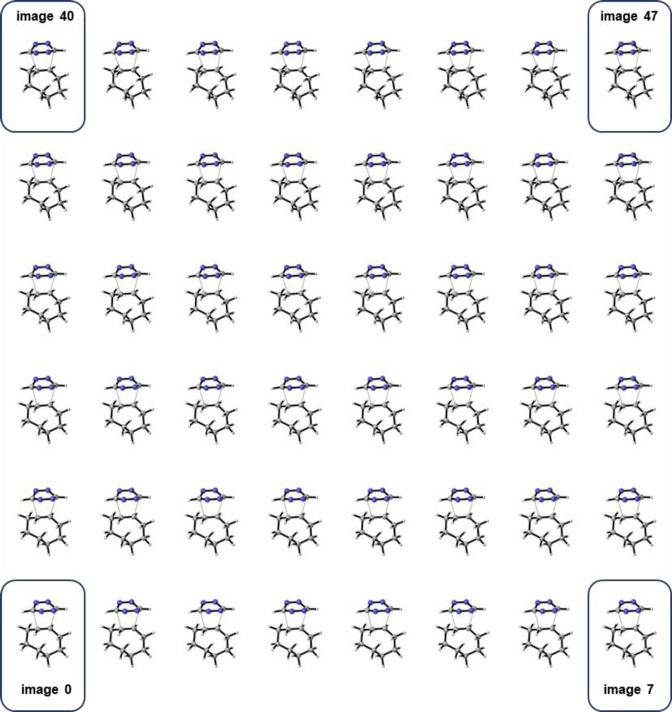
An example of a 48‐view “multi‐view image” depicting a transition state, generated using CYLview. This demonstrates a format suitable for use with autostereoscopic displays.

Creating custom content for these displays is often straightforward. Typically, one must supply images or videos where different perspectives are arranged in a unique pattern, suitable for autostereoscopic rendering, as exemplified by the 48‐view “multi‐view image” in Figure 3. These patterns serve as inputs that the monitor then uses to transform into the correct 3D visualization. For instance, a simple Python script can convert POVray files produced by CYLview 1.0, a prominent tool in computational organic chemistry, into such patterns, resulting in high‐quality images of 3D structures.^[6]^

## Use in Computational Chemistry Research

**Figure 4:**
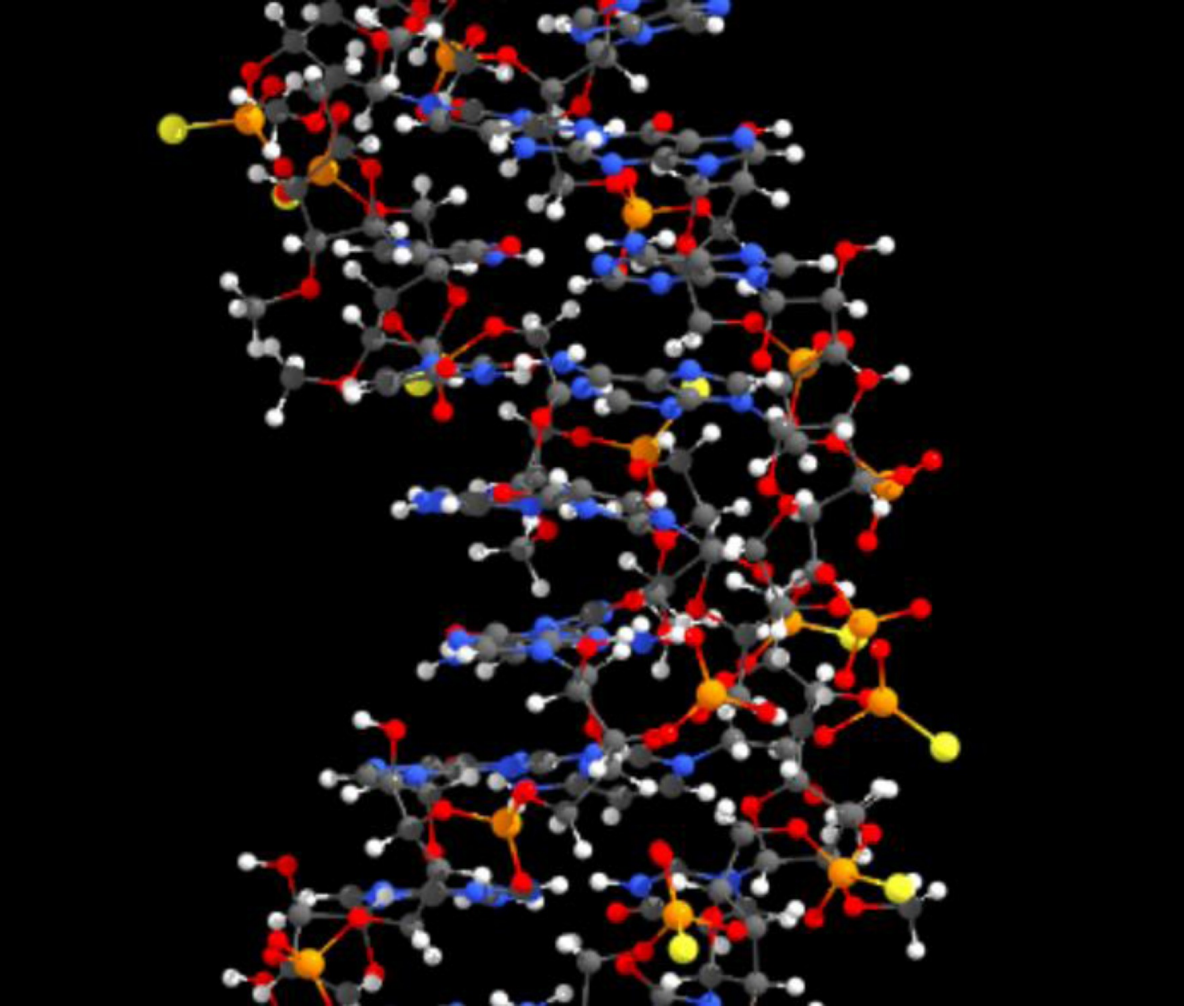
3D representation of a segment of an RNA double helix, illustrating the complexity and heavy overlap of atoms, which can make understanding the structure challenging.

In one of our current projects, a PhD student faced the challenge of modifying specific atoms in an RNA double helix consisting of approximately 1000 atoms (Figure 4). Unsurprisingly, she struggled due to the heavy overlap of different structural parts and atoms. Understanding the 3D arrangement requires constant rotation of the structures and observation of the relative movement of atoms. This process is mentally taxing and prone to errors. Using autostereoscopic displays to provide a 3D view of the structure has the potential to significantly reduce the effort, time, and error rate when working with complex structures like this.

For seasoned computational chemists handling relatively smaller systems, the challenges in understanding the 3D configuration might be less pronounced. However, even for these experienced chemists, complex structures with extensive overlapping atoms, as depicted in Figure 4, can be particularly challenging to navigate and interpret. Therefore, the importance of a good 3D visualization should not be underestimated. Integrating autostereoscopic displays within established workflows can proffer distinct advantages such as accelerated 3D structural identification. Particularly, for those new to the field or less experienced, these displays can be invaluable. In this realm, single‐view systems equipped with eye‐tracking present a seamless and economically viable integration, bridging the gap between advanced technology and real‐world practicality.

Additionally, using autostereoscopic displays can improve the communication of computational results to colleagues from other areas, as it removes the need for them to be experienced in translating 2D representations into mental 3D models. In this context, the collaborative nature of multi‐view displays would play an important role.

Incorporating multi‐view autostereoscopic displays into scientific presentations can enhance the communication of complex 3D structures, facilitating audience understanding of molecular spatial relationships. This can be particularly beneficial when presenting research findings to colleagues with diverse scientific backgrounds.

## Use in Teaching and Science Communication

Autostereoscopic displays offer several advantages in the realm of teaching and science communication, making complex 3D molecular structures more accessible and engaging for students and the general public. The following uses case are examples of areas where this technology can provide significant advantages over conventional solutions.

### Enhancing classroom learning

Implementing multi‐view autostereoscopic displays in classrooms can provide students with an interactive and engaging way to learn about molecular structures. By observing dynamic 3D representations, students can develop a better understanding of the spatial relationships between atoms in a molecule.^[7]^ This can be particularly advantageous in workshop or laboratory settings, where students collaborate to analyze and discuss molecular structures.

### Facilitating group work and discussions

Multi‐view autostereoscopic displays enable multiple users to experience the 3D content simultaneously, fostering collaborative learning and problem‐solving among students. This can be especially beneficial in workshops or laboratory settings, where students can work together to analyze and discuss molecular structures.

### Demonstrating molecular dynamics

Unlike static 3D models or 3D printed structures, autostereoscopic displays can visualize dynamic molecular structures. This allows for a more comprehensive understanding of molecular interactions and mechanisms, such as protein folding, molecular docking, or chemical reactions.

### Science outreach and public engagement

Autostereoscopic displays can serve as an effective tool for science outreach and public engagement, captivating the audience‘s attention with the “wow effect” of the 3D representations. By highlighting the intersection of chemistry, computer science, and engineering, these displays can spark curiosity and interest in scientific concepts, fostering interdisciplinary thinking.

## Strengths and Limitations

The notable strength of autostereoscopic displays lies in their ability to present accurate 3D representations of molecular structures with depth and parallax. This fosters a more intuitive grasp of the spatial relationships between atoms. Importantly, there is no need for users to wear specialized glasses or headsets, ensuring a smooth and comfortable viewing experience. Additionally, these displays can dynamically visualize molecular structures, giving a deeper understanding of molecular interactions and mechanisms when compared to static 3D models or 3D printed structures.

### Multi‐view autostereoscopic displays

A significant advantage of multi‐view autostereoscopic displays is their capability to offer multiple users simultaneous access to the 3D content. This promotes collaborative learning and problem‐solving among students, researchers, and interdisciplinary teams. Nonetheless, there are challenges. Their current high cost may constrain their widespread use in educational and research environments. While the expectation is that affordability will increase with continued technological evolution, the question of their adoption remains, especially with competing emerging technologies. The requirement to produce multiple views for each user means that the resolution per view in multi‐view autostereoscopic displays is significantly reduced relative to conventional 2D displays, potentially compromising the visual clarity and intricacy of the 3D structures displayed. The need to generate these multiple perspectives also demands significant computational resources, possibly restricting their application in resource‐limited settings. Finally, despite providing a spectrum of perspectives, the viewing angle remains narrower than the immersive experience given by VR headsets, necessitating users to stay within the stipulated viewing zone to fully benefit from the 3D representation.

### Single‐view autostereoscopic displays

Shifting focus to single‐view autostereoscopic displays, they favor higher resolution, decreased computational demands, and a considerably reduced cost over the capacity for multiple simultaneous viewers. Impressively, even the first‐generation commercial models are reasonably priced. The primary limitation here is the singular user experience for the 3D content. Yet, for many applications, this limitation is inconsequential, positioning them as an optimal choice for scenarios that do not mandate multi‐viewer engagement. Presently, however, the software support for these displays does not match that of multi‐view autostereoscopic displays or certain alternatives like VR headsets.

## Alternatives

While autostereoscopic displays offer numerous benefits for chemistry education and research, there are alternative technologies available, each with its own set of advantages and disadvantages. Some of these alternative solutions include:

### Stereoscopic displays

Stereoscopic displays, such as those using active shutter or polarization techniques,[^5^, ^8^] can provide a 3D representation of molecular structures when used with appropriate glasses. Although these displays can provide depth perception, they typically lack parallax and may be less immersive than multi‐view autostereoscopic displays. However, these screens are widely available and affordable.

### Holographic illusion pyramids

Holographic illusion pyramids reflect an image from a screen or projector towards the viewer, creating the appearance of a hovering 3D object using the Pepper's ghost technology.^[9]^ While this technology is visually striking and frequently used in sales or museums,^[10]^ the views displayed remain 2D, retaining the inherent limitations of 2D representations.

### Virtual reality (VR) headsets

Virtual reality headsets provide an immersive, highly accurate representation of 3D molecular structures. Users can fully interact with and manipulate the structures in a virtual environment. However, the cumbersome nature of VR headsets and their limitation to a single user at a time can restrict their usefulness in collaborative settings. Additionally, some people experience motion sickness when using such devices.

### Displays with camera viewing angles

Some displays, such as those from United Screens, use cameras to track the viewer‘s position and adjust the displayed view accordingly.^[11]^ This approach offers a parallax‐like effect, but lacks the true depth perception that multi‐view autostereoscopic displays can deliver. Additionally, it can only provide a view for one person at a time, essentially making it a single‐view system. While this technology can offer a more personalized viewing experience, it might not completely encompass the range of perspectives and depth provided by autostereoscopic displays.

### Volumetric displays

Volumetric displays create 3D images by illuminating a physical volume of space, allowing users to perceive depth and parallax without the need for special glasses. One notable application of this technology has been in medical imaging, where it has been used to visualize complex anatomical structures.^[12]^ Despite these displays′ ability to provide impressive 3D visualizations, they remain in the early stages of development and are not yet widely accessible for practical applications.

### True holographic displays

True holographic displays, based on light‐field technology, are in development with the aim to generate 3D images by recording and reconstructing the full light field of a scene or object.^[11]^ These displays have the potential to provide an immersive and accurate representation of 3D structures, offering both depth and parallax, without the need for any special glasses or headsets. While true holographic displays are still in the early stages of development with no actual products widely available, they hold significant potential for future applications in chemistry education and research as the technology continues to evolve and become more accessible.

## Conclusions

Autostereoscopic displays offer immersive 3D visualization without the need for special 3D glasses or headsets. The technology‘s unique ability to depict molecular structures with depth and parallax could be a game‐changer.

Although multi‐view variants of autostereoscopic displays are still in their early stages and come with high costs, in my opinion they are likely to find their niche in specialized settings, such as mixed‐reality labs. On the other hand, single‐view autostereoscopic displays, with their already relatively affordable price and reduced computational demands, seem to be paving the way for broader adoption. Even as first‐generation products, their market pricing is competitive. They cater to individual viewers, which might be a limitation in some contexts, but for numerous applications in teaching and research, this suffices. It is worth noting, however, that these displays are in need of robust software support to unlock their full potential.

From my perspective, the promise of autostereoscopic displays, especially the single‐view variants, in the realm of pedagogy and scholarly communication is vast. They have the potential to deeply enhance educational experiences, making complex molecular structures more comprehensible and facilitating clearer discussions among researchers. Additionally, the collaborative nature of multi‐view displays allows a shared 3D experience, fostering group discussions and problem solving.

There are challenges to be addressed, including the premium pricing of multi‐view displays and their reduced resolution, as well as the intensive computational requirements. Nevertheless, the benefits that this technology could bring to the world of chemistry are undeniable. As the technology matures and becomes more widely available, many of these initial challenges might be overcome.

To sum up, multi‐view autostereoscopic displays could find their place in more specialized settings, whereas single‐view displays are better positioned for mainstream adoption, potentially reshaping our understanding and communication of 3D molecular structures in chemistry. My university‘s commitment to this technology is evident through the establishment of our new mixed‐reality lab featuring multi‐view autostereoscopic displays. This dedication is mirrored in the wider field with rapid advancements, such as the introduction of new single‐view laptops.^[3c]^ Additionally, significant backing from leading venture capitalists for multi‐view displays highlights the dynamic and promising future of display technologies.^[13]^

## Conflict of interest

The authors declare no conflict of interest.

1

## Supporting information

As a service to our authors and readers, this journal provides supporting information supplied by the authors. Such materials are peer reviewed and may be re‐organized for online delivery, but are not copy‐edited or typeset. Technical support issues arising from supporting information (other than missing files) should be addressed to the authors.

Supporting Information

Supporting Information

## Data Availability

Data sharing is not applicable to this article as no new data were created or analyzed in this study.
