# Supplementary material for: “Holographic” Autostereoscopic Displays: A Perspective on Their Technology and Potential Impact in Chemistry
Source: Chemistry. 2023 Sep 14;29(61):e202301746. doi: 10.1002/chem.202301746 (PMC10946886; doi:10.1002/chem.202301746)
Supplement: Supplementary file 1 — Supporting Information [file CHEM-29-0-s001.pdf]

Chemistry–A European Journal

Supporting Information

"Holographic" Autostereoscopic Displays: A Perspective on Their Technology and Potential Impact in Chemistry

Dennis Svatunek*

Contents

Hands-on Experiences with Autostereoscopic Display Technologies S2

Experience with Displays by Looking Glass Factory S2

References.. S3

Hands-on Experiences with Autostereoscopic Display Technologies

In the course of my continued research and engagement with novel technologies, I have been fortunate to gain first-hand experience with the development and application of autostereoscopic displays. Over the past years, I have explored a variety of single and multi-view autostereoscopic displays from a range of companies at showrooms, trade shows, and workshops. These include screens developed by ASUS,[1] Looking Glass Factory,[2] United Screens GMBH,[3] and Alioscopy,[4] each contributing distinctively to my understanding and critical assessment of the technology.

One particularly enlightening experience was my participation in a closed visual computing workshop which included a 'hackathon'. Within this setting, I had the chance to access and evaluate these displays beyond their graphic fidelity, examining their practical use. This hands-on experience was instrumental in shaping my perspective on the current state and future potential of holographic technology.

Of particular note are the screens produced by the Looking Glass Factory, a leading player in the field of multi-view autostereoscopic displays. Termed as “holographic” and “light field” displays, their products feature a lenticular lens system enabling up to 100 unique views within a 53 to 58° viewing angle. Beyond hardware, Looking Glass Factory has developed an expansive ecosystem of software and tools facilitating the use of their displays. I am in possession of one of their compact units, the Portrait, which serves as a striking exemplar of this technology in action. My experience with this display has granted me a pragmatic understanding of the capabilities, software ecosystems, creation of 3D content, and potential applications of multi-view holographic displays.

Recently, I also had the opportunity to experiment with the ASUS ProArt Studiobook 16 3D OLED.[1] This laptop incorporates a single-view autostereoscopic display, coupled with eye-tracking technology for dynamic viewing angle adjustments. Particularly, this experience reinforced my belief that this technology is here to stay and will play a significant role in future visualization methods.

The insights drawn from these practical experiences have significantly shaped the perspectives and conclusions presented in the main manuscript. The subtleties of this technology, its present limitations, and potential for future advancements are largely informed by these direct encounters. The ongoing developments and investments into this field have compelled me to share this emerging technology with my fellow chemists.

Experience with Displays by Looking Glass Factory

Looking Glass Factory pioneered the commercial availability of multi-view autostereoscopic displays. They now offer a range of displays including the compact "Portrait", as well as 32" and 65" versions. These displays utilize a lenticular lens system, allowing for up to 100 distinct views within a 53 to 58° viewing angle. Beyond the hardware, Looking Glass Factory boasts an extensive software ecosystem tailored for their displays. This includes plugins for game engines like Unreal Engine and Unity, support for the renowned 3D modeling software Blender, and generic APIs for 3D environments. Schrödinger has also integrated native support for these displays in PyMol. For those interested in crafting their own content, Looking Glass Factory introduces "Quilts", which are image or video formats where different views of an object are systematically arranged (refer to Figure 3). While exploring the software ecosystem, I devised a straightforward python-based method to generate Quilt videos and images using the CYLview molecular viewer.[5] In conclusion, I am genuinely impressed by the vast ecosystem surrounding these displays and the simplicity in creating personalized content.

References

[1] ProArt Studiobook 16 OLED (H7604), accessed: 08/08/2023, https://www.asus.com/laptops/for-creators/proart-studiobook/proart-studiobook-16-oled-h7604/

[2] Looking Glass | Holograms and Conversional AI Characters, accessed: 30/05/2023, https://lookingglassfactory.com/

[3] ZVIEW: autosterescopic eye-tracking 3D-displays for professionals, accessed: 31/05/2023, https://www.united-screens.tv/en/zview/

[4] Alioscopy | Glasses-free 3D displays, accessed: 08/08/2023, https://www.alioscopy.com/en/home.php

[5] a) CYLview Visualization Software, accessed: 30/05/2023, https://www.cylview.org/index.html; b) CYLview2LookingGlass, accessed: 08/08/2023, https://github.com/dsvatunek/CYLview2LookingGlass/releases/tag/v1.0.0
